# Comparisons of early and delayed abstainers and its effects on long-term smoking cessation in Taiwan

**DOI:** 10.1186/s13011-019-0218-1

**Published:** 2019-08-14

**Authors:** Yu-Chen Chang, Wei-Hsin Huang, Chia-Ying Tsai, Lee-Ching Hwang

**Affiliations:** 1 The Department of Family Medicine, MacKay Memorial Hospital, No.92, Sec. 2, Zhongshan N. Rd., Zhongshan Dist, Taipei City, 104 Taiwan; 2 Wanrong Township Public Health Center, Hualien County, Taiwan; 3 The Department of Medicine, MacKay Medical College, New Taipei City, Taiwan

**Keywords:** Cigarette smoking, Quitting pattern, Behavior change, 6-month point prevalence abstinence

## Abstract

**Background:**

Different quitting trajectories may reflect the stages of behavior change among smokers. The nature of quitting patterns could help the public health sector to design appropriate cessation plans. This study compared demographic, health, and behavioral characteristics and the effects of long-term abstinence between early and delayed abstainers.

**Methods:**

We retrospectively sampled 142 abstainers from smoking cessation clinic participants for a study conducted from January 1 to December 31, 2017. Baseline information was obtained at the first clinic visit, and phone interviews were conducted 2 weeks, 3 months, and 6 months later. The 7-day point prevalence abstinence was employed for measuring their quitting status. We defined early abstainers as those who attained abstinence by second week and delayed abstainers as those who had done so by the third month. We compared their characteristics and 6-month quit rates and examined potential predictors between the two quitting patterns.

**Results:**

One hundred forty-two participants were included with 87 (61.3%) early abstainers and 55 (38.7%) delayed abstainers. Early abstainers were older with more comorbidities, presenting longer smoking duration, higher exhaled carbon monoxide (CO) concentration and Fagerstrom Test of Cigarette Dependence (FTCD) scores. The 6-month abstinence rate was high for both quitting patterns with no significant difference (83.9% versus 81.8%, *p* = 0.7462). Higher FTCD scores and exhaled CO concentration were potential predictors for early abstainers with adjusted odds ratio 1.16 (95% confidence interval [CI], 1.01–1.33) and 1.04 (95% CI, 1.00–1.08) respectively.

**Conclusions:**

Our results associated early abstainers with older age, more comorbidities and higher nicotine dependence. Both groups achieved good long-term abstinence maintenance. Although early abstainers may achieve earlier reduction of health risks, smokers could still benefit from long-term abstinence if they can manage to quit smoking even at later phases of cessation courses.

## Background

Smoking has persistently been identified as one of the most critical public health issues worldwide. In Taiwan, the prevalence of smoking among adults has dropped from 21.9% in 2008 to 15.3% in 2016. However, economic losses related to tobacco use remain high at approximately 50 billion New Taiwan dollars annually. The overall quitting rate within 6 months remains low at less than 30% [1]. Numerous factors such as gender, age, nicotine dependence and perceived self-efficacy have been proposed as potential determinants for abstinence maintenance [2–5].

Different quitting patterns also influence smokers’ sustained abstinence status. Smoking is regarded as a dynamic process of behavior change, which is divided into five stages according to Prochaska and DiClementi’s transtheoretical model: 1) precontemplation; 2) contemplation; 3) preparation; 4) action; and 5) maintenance [6]. Researchers who conducted a U.S.-based study investigating different quitting patterns assumed that participants in the preparation stage at the time of recruitment were more likely to be immediate quitters, whereas those in the precontemplation or contemplation stages were prone to be delayed quitters [7]. Several studies demonstrated that early abstinence served as strong predictor for successful continuous abstinence [8–10]. In another study proposed by Hughes JR et al., smokers who chose to defer their quit date until the 5th week after initiating medication reported lower probability in achieving sustained abstinence compared with those who quit smoking earlier in the course of cessation [11].

According to the smoking cessation program in Taiwan, participants benefit from pharmaceutical reimbursement for two treatment courses annually at cessation clinics. Each course provides a maximum of 8 weeks of pharmacotherapy, professional counseling and subsidies for each clinic visit, and individual telephone follow-ups by trained counselors are offered at the third- and sixth-month points after their quit date. In our hospital, aside from regular third- and sixth-month phone interviews, an additional, earlier follow-up call was also conducted 2 weeks after smokers’ first clinic visit, which provided an opportunity to further investigate the dynamics of the quitting process. To our knowledge, although previous studies have implied the significance of quitting trajectories and their influence on successful abstinence, it remains uncertain whether similar traits and long-term effects of early and delayed abstinence also exist in Asian populations. In addition, if the hypothesis of the present study is true, and if early abstinence contributes to the successful maintenance of abstinence, Health Promotion Administration’s (HPA) incorporation of an earlier phone follow-up into the current smoking cessation program to identify this additional predictor would be of value. Different treatment or counseling strategies may also be established accordingly. Therefore, in this study, we aimed to compare the characteristics and predictors of early and delayed abstainers, as well as the effects of cessation time on long-term abstinence rates among patients at a smoking cessation outpatient clinic in Taiwan.

## Methods

### Study subjects and setting

We retrospectively reviewed the smoking cessation clinic participants in a smoke-free medical center in Northern Taiwan from January 1 to December 31, 2017. A total of 485 smokers aged over 18 years volunteered to attend the smoking cessation program. All of the participants were qualified for inclusion in Taiwan’s second-generation treatment trial program with either an FTCD score of at least 4 points and/or smoking more than 10 cigarettes per day. The subjects received medical therapy and counseling from physicians during a maximum 8-week course divided into several clinic visits. Varenicline was prescribed as first-line medical treatment during the 8-week course, and a combination of nicotine patches and gum was administered only when the subject contradicted or had been previously intolerant to varenicline. Their compliance to medication was ensured during each follow-up. The quit date was set one week after initiating medical treatment during the first clinic visit. The participants were also required to complete a questionnaire designed by our HPA. This questionnaire comprehensively collected their demographic data and smoking history.

We identified 202 participants who achieved successful abstinence at the end of third month after first clinic visit. Eventually, 142 subjects were enrolled in our analysis after excluding those who failed to complete the standard treatment course and those with missing data regarding their smoking history and related parameters. They were divided into the two groups of early and delayed abstainers. Early abstainers were defined as having achieved successful abstinence at both the end of the second week and third month after first clinic visit, whereas delayed abstainers were defined as having failed in attaining abstinence at the end of 2 weeks but having succeeded in doing so 3 months after the first clinic visit.

This study was conducted with the approval of the institutional review board of Mackay Memorial Hospital, Taipei, Taiwan (application number, 17MMHIS049). All subjects provided written informed consent to participate in the study, and their identities have been kept confidential.

### Data collection and outcome measures

The primary outcome measure was smoking cessation success at 6 months. Telephone interviews of individual participants were conducted by a professional counselor at the end of 2 weeks, 3 months, and 6 months after the participants’ first clinic visit to assess their smoking position and drug adherence. The counselor also provided additional counseling and mental support. The smoking status of each subject was defined according to the point of each follow-up by using the 7-day point prevalence abstinence. This outcome measurement has routinely been incorporated into current smoking cessation programs in Taiwan. Previous systemic reviews have indicated that prolonged or point prevalence abstinence measures are highly related and can be inter-converted with moderate accuracy [12]. Point prevalence abstinence measures encompass the advantage of attenuating memory bias and variability due to missing data. They are also capable of detecting delayed quitting in comparison to prolonged abstinence measures. Those who missed the phone follow-ups were assumed to have exhibited a failure to quit or sustain quitting status at that point in time.

All of the participants completed a questionnaire designed by the HPA that gathered information concerning their demographic characteristics, past medical history, smoking history, FTCD scores, quitting motives, and urge to smoke during their first clinic visit. The *urge to smoke* section of the questionnaire consisted of two series of questions representing intrinsic and extrinsic factors, respectively. However, this section was not validated by establishing face validity or pilot testing. Intrinsic factors included nine items signifying different psychological conditions arousing a desire or craving for smoking. For example, one of the questions was “do you want to use tobacco products when you need to concentrate on your work?” Extrinsic factors contained 19 items indicating environmental or socio-cultural factors contributing to trends in tobacco use. For example, the question “do you want to use tobacco products when there is someone smoking around you?” was used as one of the assessment items for extrinsic factors. For each component in the *urge to smoke* section, participants reported positive result by answering “yes” to any of the questions. A breath sample of carbon monoxide (CO) was also obtained upon the participants’ first clinic visit.

### Statistical analysis

Data were presented as mean ± standard deviation or as percentages. Statistical analysis was performed using SPSS version 25.0 (SPSS Inc., Chicago, IL). Categorical data were analyzed with the chi-squared test, and continuous data were analyzed with the independent samples t-test. Univariate and multivariate logistic regression analysis was applied for assessments of the association between different quitting patterns and baseline characteristics. For some categorical variables with some missing data, we only included remaining subjects for further analysis. The odds ratios and 95% confidence intervals were calculated. A two-tailed *p*-value < 0.05 was considered statistically significant.

## Results

The characteristics of demographic data are shown in Table 1. A total of 142 participants were included in our analysis, among whom nearly 70% were male and ages ranged from 34 to 61 years. 13 (9.2%) subjects missed the phone follow-ups in the second week after the first clinic visit. 2 (1.4%) subjects missed the phone interviews in the sixth month after the first clinic visit. Just over 61% of subjects were categorized as early abstainers, and nearly a third of participants reported comorbidities including malignancy, cardiovascular disease (including hypertension), cerebrovascular disease, or pulmonary disease. CO concentrations obtained at the first clinic visit ranged from 5.5 to 27.1 ppm, and highly nicotine-dependent smokers (with an FTCD score equal to or greater than 7 points) accounted for nearly 40% of subjects. The participants’ smoking years ranged from 12.2 to 37.6 years. Over 90% of participants were administered with varenicline.

**Table 1:** Demographic data of study subjects (*n* = 142)

Characteristics	
Age (years)	47.8 ± 13.2
Gender (male, %)	99 (69.7)
Marital status (Married (*n*, %), *n* = 127)	72 (56.7)
Educational level (below high school levels(n,%), n = 127)	99 (69.7)
Alcohol drinking habit (*n*,%)	41 (31.3)
Number of quit attempts in the past year (*n*,%, *n* = 141)	
None	89 (63.1)
1	34 (24.1)
≧2	18 (12.8)
Quitting reason for health	42 (29.8)
Ever jointed smoking cessation program service	26 (18.3)
Urge to smoke	
Intrinsic factors (*n*,%)	105 (73.9)
Extrinsic factors (*n*,%)	123 (86.6)
Comorbidities (*n*,%)	45 (31.7)
Body weight (kg)	70.1 ± 14.6
Daily cigarette count (stick)	20.3 ± 10.1
Exhaled CO concentration (ppm)	16.3 ± 10.8
FTCD score≧7 points (*n*,%)	54 (38.0)
Duration of smoking (years)	24.9 ± 12.7
Pharmacotherapy	
Varenicline (*n*,%)	135 (95.1)
NRT (*n*,%)	7 (4.9)
Early quitters (*n*, %)	87 (61.3)

The continuous variables were shown as mean ± SD; the categorical variables were shown as percentage; Comorbidities: Self-reported of cardiovascular disease, cerebrovascular disease, pulmonary disease or malignancy; *FTCD* Fagerström test for cigarette dependence, *CO* carbon monoxide, *NRT* nicotine replacement therapy

The demographic data comparing early abstainers and delayed abstainers are presented in Table 2. Compared with delayed abstainers, early abstainers were older (mean age 49.5 versus 45.1 years) and presented longer durations of smoking, and the proportion of comorbidities among this group exceeded that reported by delayed abstainers by just over 16%. Early abstainers exhibited higher FTCD scores than delayed abstainers by nearly an entire point, and the numbers of early abstainers classified as highly nicotine dependent exceeded those of delayed abstainers by 17.5%. There was no significant difference between early and delayed abstainers (83.9% versus 81.8%, *p* = 0.7462) with respect to their prevalence of abstinence at the 6-month point.

**Table 2:** Characteristics between early abstainers and delayed abstainers

Characteristics	Early abstainers *n* = 87 (61.3)	Delayed abstainers *n* = 55 (38.7)	*p* value
Age (years)	49.5 ± 13.4	45.1 ± 12.4	0.0469
Gender (male, %)	60 (69.0)	39 (70.9)	0.806
Marital status (Married (*n*, %), *n* = 127)	47 (58.8)	25 (53.2)	0.83
Educational level (below high school levels (*n*,%), *n* = 127)	61 (70.1)	38 (69.1)	0.8971
Alcohol drinking habit (*n*,%)	24 (29.4)	17 (34.0)	0.6002
Number of quit attempts in the past year (*n*,%, *n* = 141)			0.461
None	54 (62.8)	35 (63.6)	
1	23 (26.7)	11 (20.0)	
≧2	9 (10.5)	9 (16.4)	
Quitting reason for health (*n*,%)	21 (24.4)	21 (38.2)	0.0813
Urge to smoke			
Intrinsic factors (*n*,%)	68 (78.2)	37 (67.3)	0.1499
Extrinsic factors (*n*,%)	75 (86.2)	48 (87.3)	0.8558
Comorbidities (*n*,%)	33 (37.9)	12 (21.8)	0.0444
Body weight (kg)	68.6 ± 13.6	72.4 ± 15.8	0.1348
Daily cigarette count (stick)	21.2 ± 10.7	18.9 ± 9.1	0.2067
Exhaled CO concentration (ppm)	17.3 ± 11.5	14.7 ± 9.6	0.1763
FTCD score (point)	6.2 ± 2.5	5.3 ± 2.4	0.034
FTCD score≧7 points (*n*,%)	39 (44.8)	15 (27.3)	0.0358
Duration of smoking (years)	26.8 ± 12.3	22.0 ± 12.9	0.0278
6th month point prevalence abstinence (*n*, %)	73 (83.9)	45 (81.8)	0.7462

The continuous variables were shown as mean ± SD; the categorical variables were shown as percentage; Comorbidities: Self-reported of cardiovascular disease, cerebrovascular disease, pulmonary disease or malignancy; *CO* carbon monoxide, *FTCD* Fagerström test for cigarette dependence

Table 3 demonstrates the potential predictors for early abstainers identified by performing univariate and multivariate logistic regression analysis. Older age, higher FTCD scores, coexistent comorbidities and duration of smoking appeared to be predictors for turning into early abstainers. After adjusting for age and gender, it can be seen that higher FTCD scores and CO concentration at the participants’ first clinic visit contributed significantly to the tendency for becoming early abstainers.

**Table 3:** Univariate and multivariate logistic regression analysis for the predictors of early abstainers

Variables	Univariate		Multivariate	
	OR (95% CI)	*p* value	OR (95% CI)	*p* value
FTCD score	1.16 (1.01–1.33)	0.0369	1.16 (1.01–1.33)	0.0430
Comorbidities	2.19 (1.01–4.74)	0.0468	1.72 (0.71–4.19)	0.2337
Duration of smoking	1.03 (1.00–1.06)	0.0303	1.03 (0.98–1.08)	0.2574
Exhaled CO concentration	1.02 (0.99–1.06)	0.1794	1.04 (1.00–1.08)	0.0491
Intrinsic factors	1.74 (0.82–3.72)	0.1521	2.01 (0.91–4.40)	0.0829
Extrinsic factors	0.91 (0.34–2.48)	0.8558	1.01 (0.36–2.81)	0.9876
Number of quit attempts in the past year	0.91 (0.56–1.45)	0.681	0.97 (0.60–1.58)	0.902
Age	1.03 (1.00–1.06)	0.0534		

Multivariate logistic regression analysis was adjusted for gender and age. *OR* odds ratio, *95% CI* 95% confidence interval. *FTCD* Fagerström test for cigarette dependence; Comorbidities: Self-reported of cardiovascular disease, cerebrovascular disease, pulmonary disease or malignancy; *CO* carbon monoxide

## Discussion

This study compared demographic, health, and behavioral characteristics and long-term quit rates between early abstainers and delayed abstainers, as well as potential predictors of smoking cessation patterns. Early abstainers were associated with older age, more comorbidities, longer duration of smoking years, and higher nicotine dependence compared with delayed abstainers. The effect sizes were relatively small but of significance. In addition, the 6-month point prevalence abstinence rates were high among both groups with no statistical difference.

### The 6-month point prevalence abstinence rate

In contrast to previous results, there was no significant difference between early abstainers and delayed abstainers in 6-month point prevalence abstinence rates in our study. Ferguson et al. demonstrated that smokers who were abstinent at 2 weeks exhibited a higher probability of quitting maintenance until 10 weeks [8]. Similarly, the findings of Kenford et al. indicated that early abstinence during the first 2 weeks served as a strong predictor of smoking cessation at the 6-month follow-up point [9]. However, the participants of these studies used the nicotine replacement therapy to support smoking cessation. Other studies administering treatment with varenicline reported that delayed quitters were less likely to remain abstinent in 1-year follow-up; however, in these cases, the extended varenicline therapy was applied up to 12 weeks [13, 14]. In contrast, over 90% of our participants were administered with varenicline. And an 8-week course of pharmacotherapy was employed in our study. In addition, previous researches rarely reported statistically measured associations between smoking cessation patterns and health characteristics such as comorbidities or nicotine dependence levels. For example, the study by Gonzalez et al. identified all of its subjects as being “generally healthy” [13]. However, a considerable proportion of the participants in our study demonstrated high nicotine dependence and several comorbidities. Furthermore, prior studies indicated demographic and intercultural differences with respect to the stages of smoking behavior change among different countries, with the highest rates during the precontemplation stage reported among Chinese subjects [15]. Cultural differences in self-efficacy and behavior transition could be a potential determinant of abstinence maintenance among early and delayed abstainers.

Although we did not observe a significant difference between early abstainers and delayed abstainers regarding long-term quitting rates, the 6-month point prevalence abstinence rate was distinctly high for both groups in our study. According to the Taiwan Tobacco Control Annual Report 2017, the 6-month point quitting rate was only 35.0% among general smokers receiving treatment at medical centers [1]. Previous researches implied that recent success or failure in quitting smoking may substantially affect individuals’ self-efficacy level [16–18]. Additionally, evidence indicated patients with clinical manifestations of chronic illness may also present higher levels of self-efficacy [19, 20]. The subjects in our study attained success in their quit attempts at varying time points. They also reported higher proportion of major comorbidities such as cardiovascular or cerebrovascular disease compared with subjects in other researches. Hence, they might demonstrate higher health-related motivations in maintaining abstinence compared with general smokers. Furthermore, the frequent clinic and phone follow-up sessions conducted in our smoke-free hospital also provided behavioral supports that improved the success rate of smoking cessation among these participants. A Taiwanese study found that total number of clinic visits was associated with higher abstinence rates [21].

### The effect of age, nicotine dependence and craving

Previous studies have indicated that older age was a potential predictor for successful smoking cessation [22, 23], and two Japanese investigations related a younger age at quit attempt to increased risk of lapse and relapse [19, 24]. In our study, the average age of early abstainers was significantly older than that of delayed abstainers, which may explain the longer duration of smoking years and higher nicotine dependence among this group. In addition, early abstainers reported a higher proportion of chronic illness. They may have higher levels of self-efficacy to engage in health behavior change activities, which could enhance their motivation to quit smoking at an earlier period.

The evidence regarding negative impacts of nicotine dependence on smoking cessation is robust but not fully consistent in previous researches [3, 23, 25]. FTCD scores have been demonstrated to be a reliable indicator for the severity of withdrawal symptoms, and exhaled CO concentration levels correlate to daily cigarette counts [26, 27]. Both outcome measures were applied for assessing nicotine dependence in this study. In the International Tobacco Control Four-Country Survey using The Heaviness of Smoking Index for nicotine dependence evaluation, Yong et al. found its predictive value to be limited to short-term smoking relapses [28]. In one Japanese study using varenicline as pharmaceutical therapy, FTCD scores were non-significant between relapsers and non-relapsers [24]. In our study, the participants with higher FTCD scores and higher exhaled CO levels were prone to become early abstainers after adjusting for several factors. The study design and sampling process may have led to a potential collider bias and caused this inconsistent finding compared with previous results in the general population [29, 30]. However, our study focused on recent abstainers attending smoking cessation clinics in major healthcare institutions. The characteristics of these subjects are distinct because they may have more comorbidities and higher health beliefs. Thus, the presumed impact of nicotine dependence in this group could be varied or even attenuated.

Previously conducted studies identified cravings as a potential barrier to initiating quit attempts and a predictor for relapse [31, 32]. However, inconsistent results have been presented because of diverse conceptual frameworks regarding the definition of cravings [33, 34]. Our results did not observe a significant difference between early and delayed abstainers in either intrinsic or extrinsic factors affecting urge to smoke. This could be explained by a relative low answer rate in this part of the questionnaire and our measurement instrument was not validated. Past researches proposed that several brief and reliable multiple-item scales or the single craving item could be utilized to assess craving [33]. Similarly validated tools should be considered for modification of our questionnaire. In addition, past studies found that varenicline may reduce craving and the rewarding effects of tobacco more than other medications [35, 36]. The influence of smoking urges could have been attenuated by the administration of varenicline to most of our study participants.

### Strength and limitations

This study is the first to investigate the characteristics of differing quitting patterns in Asian populations. Early abstainers successfully enter the advanced stages of action and maintenance. Hence, they should be encouraged to maintain their current healthy lifestyle, and steps should be taken to prevent them from relapsing into the smoking habit. Individuals who fail to attain early abstinence may become delayed abstainers and achieve long-term abstinence. Therefore, their health awareness must be increased, and their medical compliance should be reinforced to help them progress into the advanced stages of smoking cessation.

There are several limitations to our study. First, the relatively small sample size and the data collection from a single center might have reduced the statistical power of our results and limited their generalizability. Second, the self-reported nature of measures such as past medical history, smoking history, medical adherence and abstinence during outpatient clinic and phone follow-ups might have contributed to recall bias to a certain extent. Third, the 7-day point prevalence abstinence employed in our study is not as stable a measure as continuous abstinence rates because they emphasize only the minimum duration of abstinence. We could not obtain the detailed quitting trajectories of our participants between follow-ups. However, the 7-day point prevalence abstinence was still a suitable surrogate for subjects’ sustained abstinence status based on previous studies. Additional biochemical verifications for smoking status such as exhaled CO or saliva cotinine assessments might help improve the accuracy of such measures. Fourth, we counted missing the phone follow-up interviews as failure to quit; however, the actual smoking status of these missing subjects was uncertain. Fifth, although intrinsic and extrinsic factors were considered in our analysis, this section of questionnaire was not validated. However, this questionnaire developed by HPA is widely administered nationwide. Its reliability and validity are expected to be comprehensively established in future researches.

## Conclusions

In summary, early abstainers were associated with older age, more comorbidities, longer duration of smoking years, and higher nicotine dependence than delayed abstainers. 6-month point prevalence abstinence rates were notably high among both groups with no statistical difference. More prospective investigations addressing variable influences on quitting rates of longer duration are warranted.

## Data Availability

The datasets generated and/or analysed during the current study are not publicly available but are available from the corresponding author on reasonable request.

## Abbreviations

CO: Carbon monoxide
FTCD: Fagerstrom Test of Cigarette Dependence
HPA: Health Promotion Administration
